# Aggressive Progression of High Programmed Death-Ligand 1 (PD-L1) Non-small Cell Lung Cancer Presenting as Life-Threatening Esophageal Obstruction: A Case of Food Impaction Secondary to Subcarinal Lymph Node Compression

**DOI:** 10.7759/cureus.86785

**Published:** 2025-06-26

**Authors:** Niroshan Ranjan, Yaman Dalati, Vidushan Sabanathan, Thanujan Thangadurai

**Affiliations:** 1 Internal Medicine, Henry Ford Health System, Jackson, USA; 2 Anesthesiology, Henry Ford Hospital, Detroit, USA; 3 Medicine, American University of the Caribbean School of Medicine, Cupecoy, SXM; 4 Family Medicine, Good Samaritan University Hospital, West Islip, USA

**Keywords:** esophageal obstruction, immunotherapy resistance, nsclc, pd-l1 expression, subcarinal lymph node

## Abstract

Dysphagia secondary to esophageal obstruction is a rare but clinically relevant presentation in the setting of non-small cell lung cancer (NSCLC). While pembrolizumab demonstrates efficacy in metastatic NSCLC with high programmed death-ligand 1 (PD-L1), diagnostic challenges in distinguishing pseudoprogression from true progression and paradoxical disease progression pose a clinical challenge, highlighting complexities inherent in immune checkpoint inhibitor resistance mechanisms. We present the case of an 82-year-old Caucasian woman with a diagnosis of stage IV NSCLC with extremely high PD-L1 expression who developed accelerated disease progression on pembrolizumab monotherapy. Following 11 cycles of immunotherapy, the patient developed life-threatening esophageal obstruction due to a massively enlarged subcarinal lymph node, causing significant extrinsic compression. This resulted in food impaction necessitating urgent endoscopic management, followed by aspiration pneumonia requiring medical intensive care unit admission. Endoscopic evaluation revealed a critically narrowed esophageal lumen with ulcerated and necrotic mucosa. To facilitate nutritional support and airway protection, a gastrostomy tube was inserted. This case highlights several key clinical points: mediastinal lymphadenopathy can result in life-threatening esophageal compression requiring immediate intervention; high PD-L1 expression level is no guarantee of immunotherapy efficacy and may paradoxically be associated with aggressive disease progression; tissue sampling is imperative to differentiate between true progression versus pseudoprogression; and gastrostomy tube insertion is a vital palliative intervention for malignant esophageal obstruction secondary to extrinsic compression.

## Introduction

Non-small cell lung cancer (NSCLC) accounts for about 85% of all lung cancer. Pembrolizumab monotherapy has emerged as a standard therapy for metastatic tumors with ≥50% programmed death-ligand 1 (PD-L1) expression levels [1]. Pembrolizumab is a PD-1 blocking antibody that works by reactivating the anti-tumor immune response, which is often suppressed in cancer. The requirement for PD-L1 expression of 50% or greater is based on clinical trial data, such as the KEYNOTE-024 study, which demonstrated superior outcomes (overall survival and progression-free survival) in this biomarker-selected patient population compared to platinum-based chemotherapy [1]. This has significantly modified the treatment landscape for advanced NSCLC.

Dysphagia in lung cancer patients is relatively uncommon, typically resulting from direct tumor invasion, radiation-induced fibrosis, or, rarely, extrinsic compression from enlarged mediastinal lymph nodes [2]. The subcarinal lymph node (station 7) is anatomically positioned anterior to the mid-esophagus and posterior to the carina. This close proximity means that expansion of these lymph nodes, particularly posteriorly due to mediastinal constraints, risks critical esophageal compression. While such mechanical obstruction is rare, it can lead to significant clinical complications.

This case report describes the aggressive progression of high PD-L1 NSCLC presenting as life-threatening esophageal obstruction, highlighting the paradoxical nature of immunotherapy resistance and the critical importance of recognizing mechanical complications from mediastinal disease progression. Our clinical question is as follows: How can we better differentiate between true progression and pseudoprogression in patients with high PD-L1 NSCLC on pembrolizumab, especially when faced with acute mechanical complications such as esophageal obstruction?

## Case presentation

A white Caucasian, 82-year-old female with a history of type 2 diabetes mellitus, hypertension, hypothyroidism, stage III HER2+ breast cancer (for which she had a lumpectomy with radiation over a decade ago), obstructive sleep apnea, major depressive disorder, and a former smoker presented with a new diagnosis of NSCLC.

Initial presentation and diagnosis

**Figure 1:**
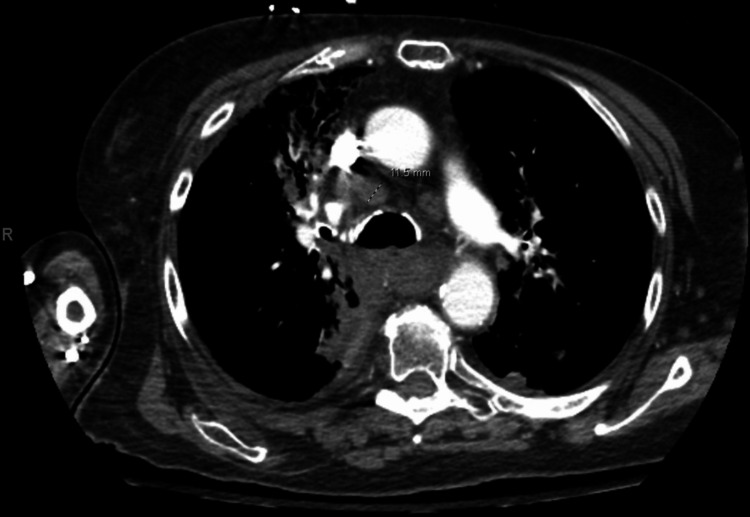
CT scan showing precarnial/right paratracheal lymph node, largest measuring 1.2 cm and showing esophageal dilation with layering debris proximal to the level of subcarinal lymph node compression. Airspace opacities noted.

At initial presentation, endobronchial ultrasound (EBUS) revealed malignant cells in subcarinal lymph node station 7. Subsequent positron emission tomography demonstrated a large fluorodeoxyglucose (FDG)-avid subcarinal mass with right-sided rib lesions concerning for metastatic disease (Figure 1). Brain magnetic resonance imaging was negative for metastases. Comprehensive molecular testing revealed exceptionally high PD-L1 expression (90%) with negative next-generation sequencing for actionable mutations, establishing a diagnosis of stage IV NSCLC suitable for first-line pembrolizumab monotherapy.

Early treatment course

Pembrolizumab monotherapy was initiated one month after an initial diagnosis. Treatment was complicated with an episode of severe cutaneous rash consistent with an immune-related adverse event, complicated by the patient's refusal to take oral steroids due to fear of insomnia. Positron emission tomography two months after therapy was notable for potential pseudoprogression, with continuation of immunotherapy with close monitoring.

Treatment complications

During the third and fourth months of treatment, the patient experienced episodes of symptomatic hypotension, near-syncope, and progressive weight loss requiring multiple emergency department visits. Despite these concerning symptoms, clinical improvement was observed by the fifth month of treatment. Serial computed tomography scans at six and seven months demonstrated improvement in thoracic lymphadenopathy, providing radiographic evidence supporting immunotherapy continuation and suggesting the earlier imaging findings represented pseudoprogression rather than true disease progression.

Disease progression

**Figure 2:**
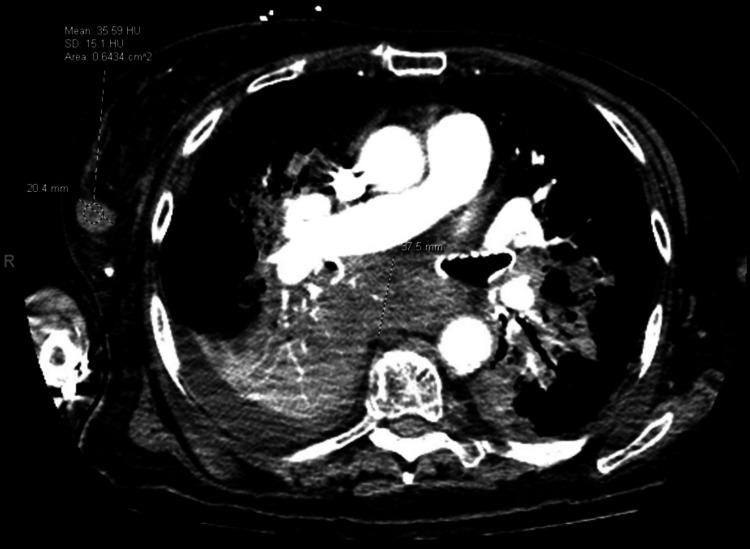
Subcarinal adenopathy measuring approximately 3.8 cm in short axis. Interval development of small right and left pleural effusion and right lower lobe (RLL) consolidation.

**Figure 3:**
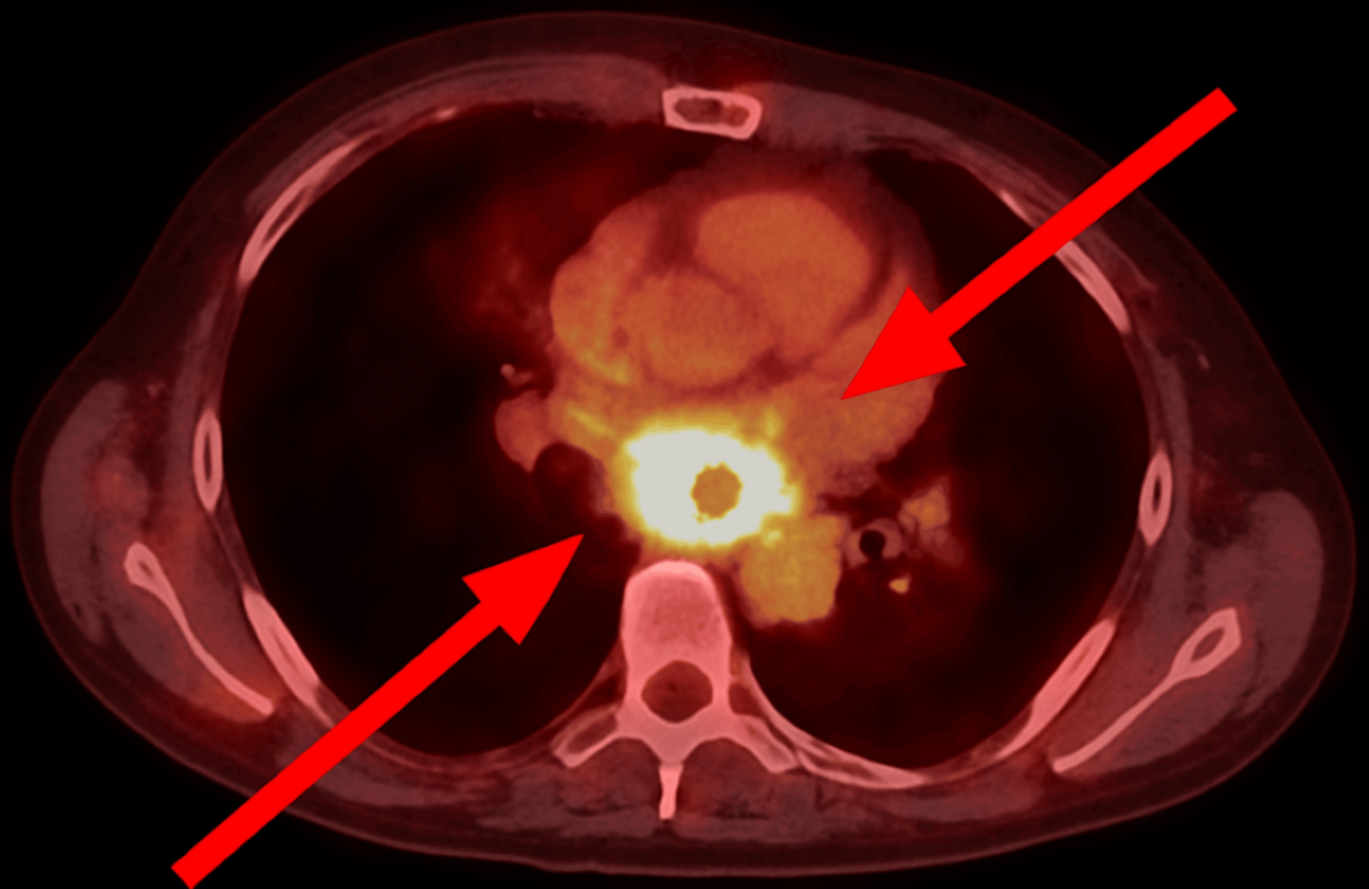
PET/CT showing increased metabolism of the mediastinal lymphadenopathy (SUV max 32.9) with central photopenic defect suggestive of necrosis.

Ten months into treatment, imaging revealed a slight increase in lymphadenopathy, though the patient remained clinically stable. She completed her eleventh cycle of pembrolizumab at eleven months, after which treatment was held due to the recurrence of the immune-related cutaneous rash. One year after initial diagnosis, restaging scans demonstrated clear cancer progression, particularly involving the subcarinal lymph node measuring 3.8 cm in short axis (Figure 2), with PET/CT revealing increased metabolic activity (SUV max 32.9, increased from previous 27.4) and central necrosis (Figure 3).

Acute clinical deterioration

**Figure 4:**
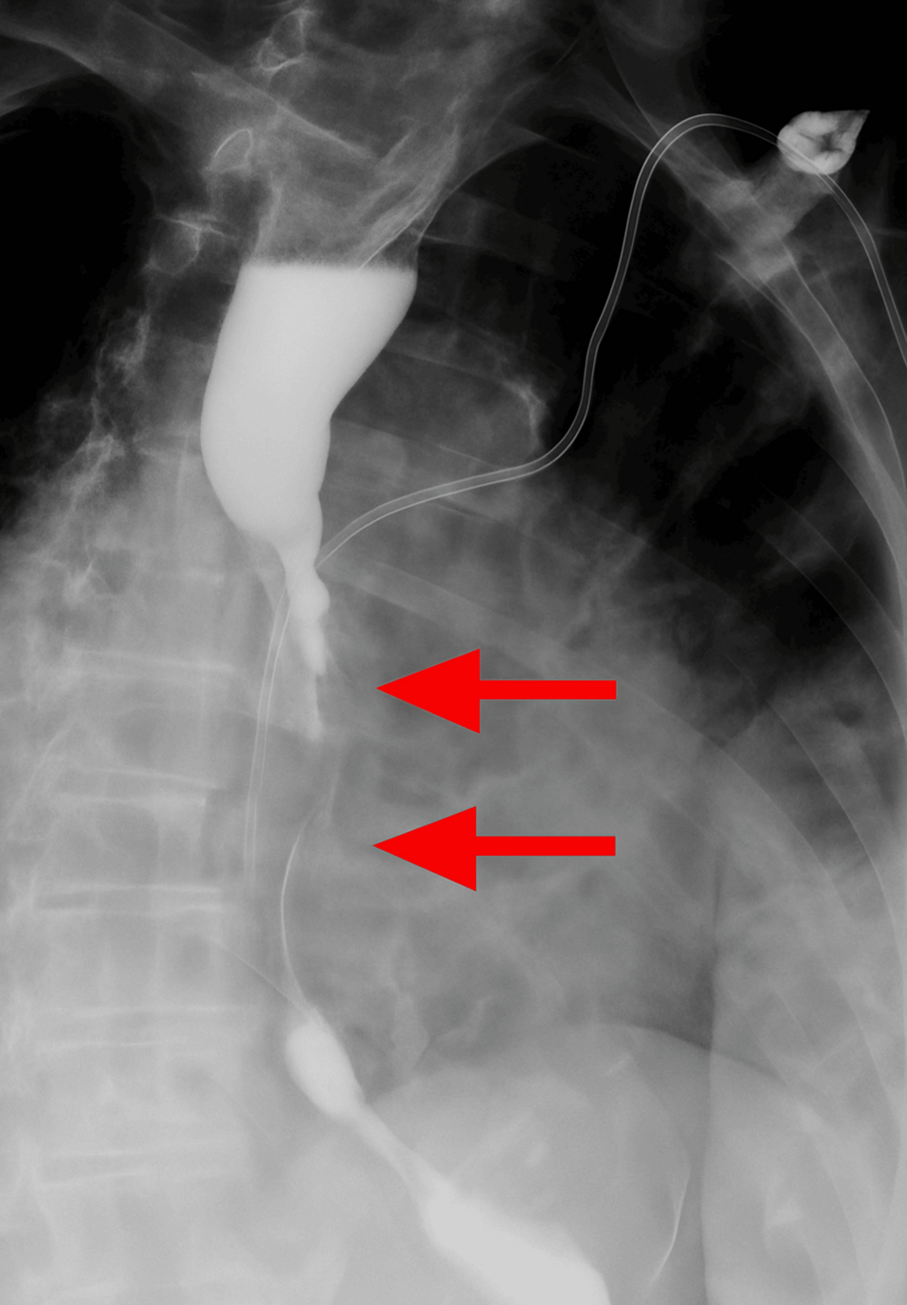
Esophagram demonstrating a filling defect causing fixed luminal narrowing in the mid to distal esophagus, measuring up to 6 cm in craniocaudal dimension.

**Figure 5:**
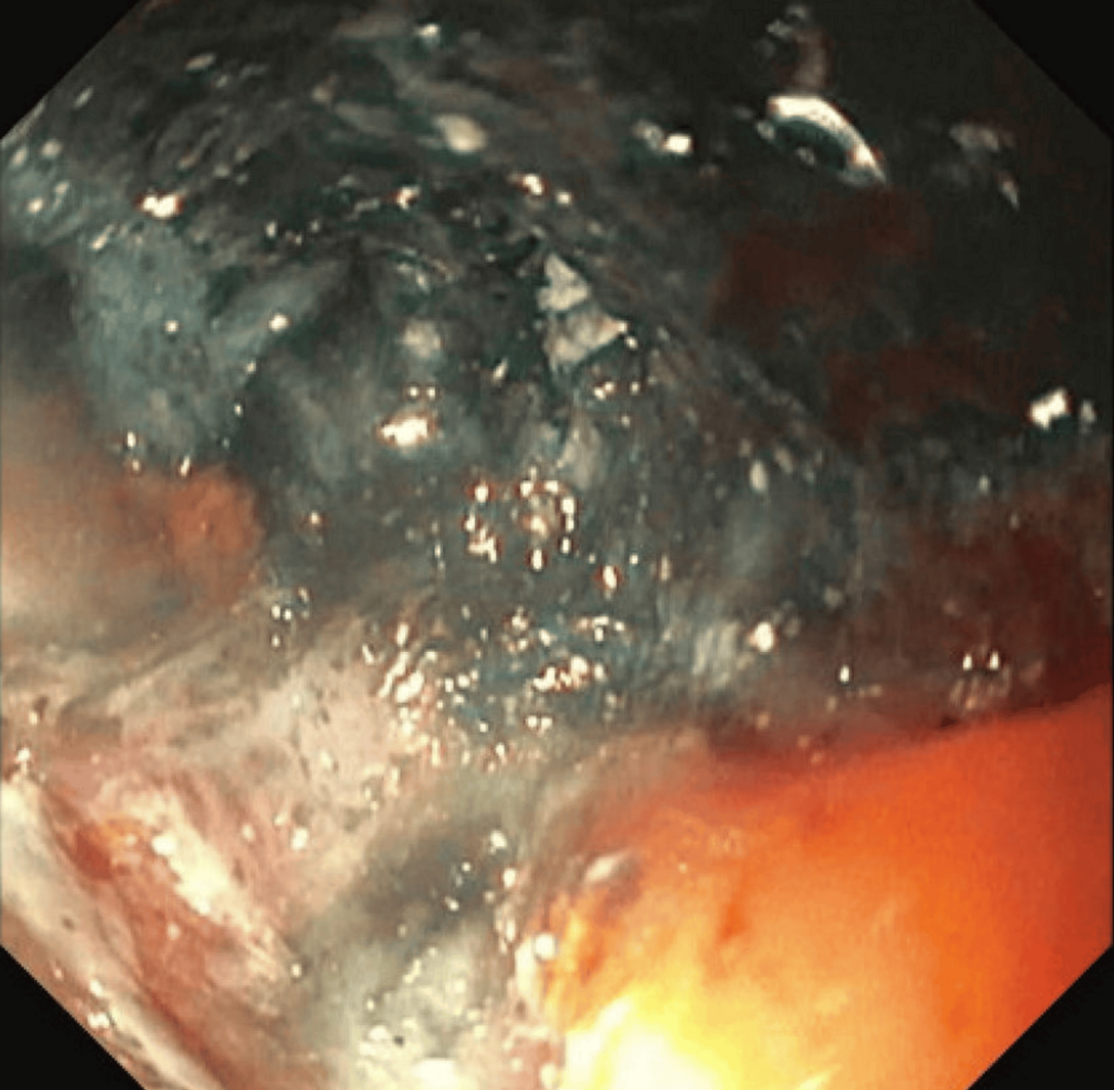
Severe extrinsic stenosis at the level of the subcarinal lymph node and ulcerated and necrotic esophageal mucosa due to severe extrinsic compression in 2/3 of the esophagus.

**Figure 6:**
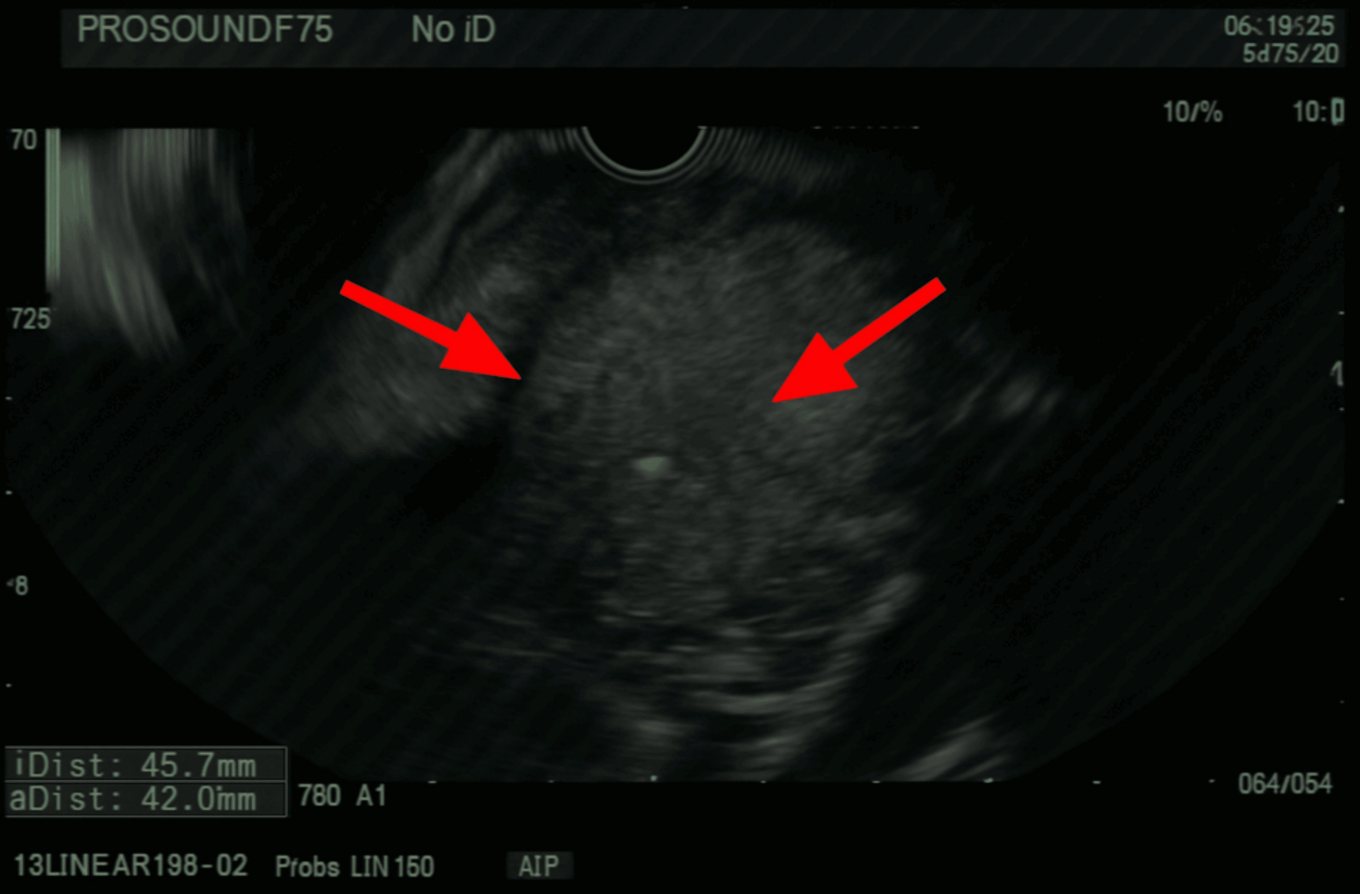
Endosonography showing a malignant-appearing lymph node in the subcarinal mediastinum, measuring 45 mm x 49 mm.

Thirteen months after initial diagnosis, the patient underwent elective esophagogastroduodenoscopy due to progressive dysphagia symptoms. This procedure revealed food impaction at 27 cm from the incisors caused by severe extrinsic compression from the markedly enlarged subcarinal lymph node. Esophagram demonstrated fixed luminal narrowing measuring 6 cm in craniocaudal dimension (Figure 3). Endoscopic evaluation showed severe stenosis with a critically narrowed lumen, measuring only 5 mm in diameter over a 4 cm length, accompanied by ulcerated and necrotic esophageal mucosa affecting approximately two-thirds of the circumference (Figures 4-6). Endoscopic ultrasound confirmed a malignant-appearing lymph node in the subcarinal mediastinum measuring 45 mm × 49 mm in maximal cross-sectional diameter (Figure 6).

Critical care management

Following the endoscopic intervention, the patient developed fever (maximum: 102.9°F), tachycardia, and progressive respiratory distress consistent with aspiration pneumonia, necessitating transfer to the medical intensive care unit. She required heated high-flow nasal cannula oxygen therapy (40 L/min, FiO₂: 80%) to maintain oxygen saturation above 95%. Vital signs revealed persistent tachycardia (130-140 beats per minute) with relatively stable blood pressure. Bedside ultrasonography demonstrated an inferior vena cava collapsibility index of 1.5 without evidence of right ventricular strain, suggesting adequate volume status without acute cor pulmonale. Physical examination revealed significantly decreased breath sounds in the left lung field.

Treatment and recovery

The patient was diagnosed with sepsis secondary to aspiration pneumonia and treated with appropriate antimicrobial therapy. Following successful management of the respiratory failure and sepsis, interventional radiology-guided gastrostomy tube placement was performed to ensure safe nutritional access and prevent future aspiration events. After clinical stabilization and completion of antibiotic therapy, the patient was discharged home with gastrostomy tube feeding support and appropriate palliative care measures.

## Discussion

This case presents a compelling illustration of the complex challenges encountered in contemporary NSCLC management, particularly highlighting the paradoxical nature of immunotherapy resistance despite optimal biomarker profiles and the rare but life-threatening complication of esophageal obstruction from mediastinal disease progression. The clinical trajectory of this 82-year-old patient underscores several critical aspects of advanced NSCLC care that warrant detailed examination.

Rarity and mechanisms of esophageal obstruction in NSCLC

Esophageal obstruction secondary to mediastinal lymphadenopathy is a clinically significant, albeit infrequent, complication of non-small cell lung cancer, found in approximately 1-2% of patients with lung cancer [2]. The pathophysiology behind the condition involves anatomical closeness between the subcarinal station of the node and the mid-esophagus. According to Camidge et al., dysphagia in patients with lung cancer is most often a result of three significant mechanisms: compression of the esophagus within the mediastinum (most frequent), compression in the pharynx and upper esophagus due to cervical lymph node deposits, and radiotherapy-induced esophageal stenosis [2].

The subcarinal lymph nodes, posterior to the carina and anterior to the esophagus, render these lymph nodes extremely likely to cause compression of the esophagus upon significant enlargement. These nodes are limited from horizontal growth due to surrounding mediastinal structures and, thus, expand posteriorly, compressing directly against the surrounding esophageal wall [3]. In our case, the markedly enlarged subcarinal lymph node, measuring 45 mm × 49 mm, created severe extrinsic compression, resulting in a critically narrowed esophageal lumen of only 5 mm in diameter over a 4 cm length. This degree of stenosis represents one of the most severe cases of malignant esophageal obstruction reported in the literature, considering that the normal esophageal diameter measures up to 30 mm [3,4]. This patient's lumen was reduced to less than 17% of normal diameter, highlighting the aggressive nature of the underlying disease process [5,6].

The development of food impaction at 27 cm from the incisors, with subsequent aspiration pneumonia requiring intensive care unit admission, demonstrates the life-threatening potential of this complication. The ulcerated and necrotic appearance of the esophageal mucosa observed during endoscopy reflects the severe mechanical trauma caused by the external compression, distinguishing this presentation from intrinsic esophageal malignancy or immune-related esophagitis. Previous case reports have documented similar presentations, though the combination of such severe stenosis with high-grade PD-L1 expression and immunotherapy resistance remains exceptionally rare [7,8].

The PD-L1 paradox and immunotherapy resistance mechanisms

Of most notable in this case is the paradoxical aggressive course of disease in the context of extremely high PD-L1 expression at 90%. Single-agent pembrolizumab has been the benchmark standard as initial treatment for metastatic NSCLC with PD-L1 expression ≥50% as a result of evidence, including from the seminal KEYNOTE-024 trial, demonstrating greater overall survival and progression-free survival with pembrolizumab compared with platinum-based chemotherapy [9]. Response levels with this biomarker-identified population are generally within a 40-45% range [10], with an assumption made that higher PD-L1 expression levels are more frequently associated with successful treatment [11].

However, clinical experience has demonstrated that PD-L1 expression alone is not a sufficient predictor of a response to treatment with immunotherapy. A 21-27% initial resistance is found in patients with NSCLC with initial immunotherapy, while secondary or acquired resistance is 52-57% in initial responders [12]. These mechanisms are multifaceted, as they are tumor-intrinsic and tumor microenvironment-dependent.

Tumor-intrinsic-resistant mechanisms involve low tumor mutational burden, compromised antigen presentation machinery, alterations in the interferon-gamma pathway, and loss of tumor suppressors such as STK11/LKB1 [13]. Tumor microenvironment also plays a significant role, with immunosuppressive factors such as regulatory T cells, myeloid-derived suppressor cells, tumor-associated macrophages, and immunosuppressive cytokines all providing a supportive environment for blunting effective anti-tumor immune responses [12,13]. In this patient's case, the aggressive clinical course, despite exceptionally high PD-L1 expression (90%) and the absence of targetable genomic alterations on next-generation sequencing, suggests the involvement of these broader, less characterized resistance pathways. While specific molecular drivers of resistance were not identified, the rapid progression underscores the complex interplay of factors that can lead to immunotherapy failure, even in seemingly ideal candidates.

Hyperprogression versus pseudoprogression: diagnostic challenges

The clinical course of our patient initially raised concerns for pseudoprogression, a phenomenon occurring in 1-5% of patients treated with immune checkpoint inhibitors, characterized by initial apparent disease progression on imaging that subsequently resolves with continued treatment [14]. Pseudoprogression is thought to result from immune cell infiltration and inflammation within tumor sites, creating the radiographic appearance of disease progression while actually representing an active immune response against the tumor.

However, distinguishing pseudoprogression from true disease progression, and particularly from hyperprogressive disease, remains a significant clinical challenge. Hyperprogressive disease, defined as rapid tumor growth acceleration following immune checkpoint inhibitor initiation, has been reported in 9-13% of NSCLC patients receiving immunotherapy [15]. This phenomenon is characterized by a dramatic increase in tumor growth rate compared to pre-treatment kinetics, often accompanied by rapid clinical deterioration.

Contemporary tools for progression assessment, beyond the standard RECIST (Response Evaluation Criteria in Solid Tumors), are increasingly important. Dynamic PET kinetics, which evaluate changes in metabolic activity over time, and circulating tumor DNA (ctDNA) analysis, which can detect molecular residual disease or emerging resistance mutations, offer promising avenues for earlier and more accurate differentiation between pseudoprogression, true progression, and hyperprogression [16,17]. In our case, several factors supported the diagnosis of true progression rather than pseudoprogression. The progressive increase in SUV maximum from 27.4 to 32.9 on PET/CT imaging, the development of central necrosis within the lymph node, and, most importantly, the progressive clinical symptoms of dysphagia culminating in food impaction, all pointed toward genuine disease progression. The importance of tissue sampling to definitively distinguish between these entities cannot be overstated, as highlighted by the endobronchial ultrasound-guided fine needle aspiration findings that confirmed viable malignancy within the subcarinal node [18].

Management considerations and therapeutic implications

Therapy for esophageal malignant obstruction should be weighed judiciously depending on the etiology behind it, as well as on the patient's overall clinical status. In intrinsic esophageal neoplasms, endoscopic treatment with debulking, balloon dilatation, or stent insertion is typically successful. External compression due to masses within the mediastinum presents more specific problems, since the conventional endoscopic treatment methods are ineffective or even hazardous due to the danger of perforation or fistula [19].

In our case, the decision to proceed with gastrostomy tube placement rather than esophageal stenting was appropriate given the severe external compression, ulcerated mucosa, and risk of aspiration. Gastrostomy tubes provide a reliable means of nutritional support while bypassing the obstructed segment entirely, reducing the risk of recurrent food impaction and aspiration events [20]. This approach aligns with established guidelines for palliative management of malignant dysphagia, particularly in cases where the obstruction is caused by external compression rather than intrinsic tumor growth.

Following the resolution of the acute esophageal obstruction and aspiration pneumonia, considerations for systemic treatment reinitiation would typically involve a comprehensive reassessment of the patient's overall performance status, disease burden, and potential alternative therapeutic strategies. Given the aggressive nature of the progression on pembrolizumab, exploring other immunotherapy combinations, chemotherapy, or targeted therapies (if new actionable mutations were identified) would be crucial. However, the patient's advanced age, comorbidities, and the severity of the acute event would necessitate a careful discussion of risks versus benefits, prioritizing palliative care and quality of life.

The development of aspiration pneumonia following endoscopic manipulation is a demonstration of procedure-related intrinsic risks within patients with severe esophageal obstruction [21]. Requiring intensive care unit admission, supplementation with a high-flow nasal cannula, and subsequent broad-spectrum antibiotics highlights the potential for rapid clinical decompensation within this patient population [22]. Resolution from sepsis and respiratory decompensation, with subsequent interventional radiology-directed gastrostomy tube insertion, demonstrates utility in multidisciplinary care for complicated oncologic emergencies [23].

## Conclusions

This case demonstrates the multifaceted complexity of current-day NSCLC management, with evidence suggesting that high PD-L1 does not dictate response to immunotherapy and, in fact, is associated with aggressive tumor growth. Life-threatening evolution from esophageal obstruction secondary to compression from a subcarinal node is a dangerous but rare complication requiring emergency multidisciplinary treatment. Important clinical take-home points include the following: (1) vigilance with dysphagia symptomatology in patients with mediastinal involvement, with swift evolution from mechanical compression resulting in life-threatening obstruction; (2) knowledge that PD-L1 expression alone is not adequate for prediction of response, requiring overall assessment using biomarkers; (3) utmost value for tissue sampling in distinguishing between actual progression versus pseudoprogression whenever clinically feasible; and (4) feeding gastrostomy tube insertion as a very effective symptom palliation for obstructive esophageal cancer secondary to extrinsic compression, with nutritional relief as well as airway protection in judiciously selected patients.

Follow-up studies should focus on the discovery of new biomarkers that may improve the accuracy of response prediction to immunotherapy, especially in patients with high PD-L1 expression. Improved care pathways for managing acute mechanical complications related to advanced NSCLC should also be developed and should include the early delivery of palliative care and the use of new interventional methods. Further research on the mechanisms driving hyperprogression and acquired resistance is also crucial in developing more effective and tailored treatment approaches for these challenging patient groups.
